# Robotic Versus Open Hepatic Arterial Infusion Pump Placement for Unresectable Intrahepatic Cholangiocarcinoma

**DOI:** 10.1245/s10434-024-15127-w

**Published:** 2024-03-18

**Authors:** Britte H. E. A. Ten Haaft, Stijn Franssen, Roderick W. J. J. van Dorst, Merve Rousian, Gabriela Pilz da Cunha, Roeland F. de Wilde, Joris I. Erdmann, Bas Groot Koerkamp, Jeroen Hagendoorn, Rutger-Jan Swijnenburg

**Affiliations:** 1 Department of Surgery, Cancer Center Amsterdam, Amsterdam UMC, University of Amsterdam, Amsterdam, The Netherlands; 2 Department of Surgery, Erasmus MC Cancer Institute, Rotterdam, The Netherlands; 3 Department of Surgery, University Medical Center Utrecht, Utrecht, The Netherlands; 4 Department of Surgery, Cancer Center Amsterdam, Amsterdam UMC, Vrije Universiteit Amsterdam, Amsterdam, The Netherlands

**Keywords:** Cholangiocarcinoma, Hepatic-arterial-infusions, Pump, Surgery, Robot

## Abstract

**Background:**

Hepatic arterial infusion pump (HAIP) chemotherapy is an effective treatment for patients with unresectable intrahepatic cholangiocarcinoma (iCCA). HAIP chemotherapy requires a catheter inserted in the gastroduodenal artery and a subcutaneous pump. The catheter can be placed using an open or robotic approach.

**Objective:**

This study aimed to compare perioperative outcomes of robotic versus open HAIP placement in patients with unresectable iCCA.

**Methods:**

We analyzed patients with unresectable iCCA included in the PUMP-II trial from January 2020 to September 2022 undergoing robotic or open HAIP placement at Amsterdam UMC, Erasmus MC, and UMC Utrecht. The primary outcome was time to functional recovery (TTFR).

**Results:**

In total, 22 robotic and 28 open HAIP placements were performed. The median TTFR was 2 days after robotic placement versus 5 days after open HAIP placement (*p* < 0.001). One patient (4.5%) in the robotic group underwent a conversion to open because of a large bulky tumor leaning on the hilum immobilizing the liver. Postoperative complications were similar—36% (8/22) after robotic placement versus 39% (11/28) after open placement (*p* = 1.000). The median length of hospital stay was shorter in the robotic group—3 versus 5 days (*p* < 0.001). All 22 robotic patients initiated HAIP chemotherapy post-surgery, i.e. 93% (26/28) in the open group (*p* = 0.497). The median time to start HAIP chemotherapy was 14 versus 18 days (*p* = 0.153).

**Conclusion:**

Robotic HAIP placement in patients with unresectable iCCA is a safe and effective procedure and is associated with a significantly shorter TTFR and hospital stay than open HAIP placement.

**Supplementary Information:**

The online version contains supplementary material available at 10.1245/s10434-024-15127-w.

Intrahepatic cholangiocarcinoma (iCCA) is a malignant neoplasm arising from the epithelial cells of the intrahepatic bile ducts. The highest incidence rates are reported in certain regions of East Asia and ranges from 10 to 71 per 100.000.^1^ To date, iCCA accounts for 20–30% of primary liver malignancies and approximately 25% of all cholangiocarcinomas.^2^

Since most patients present with locally advanced or metastatic disease as a result of aggressive tumor biology combined with late onset of symptoms, resection is only feasible in 15–30% of patients.^3^ The prognosis in the palliative setting is poor, with a median overall survival (OS) of 17 months.^4^ Standard palliative treatment for these patients consists of gemcitabine, cisplatin plus durvalumab, as indicated by the TOPAZ clinical trial.^5^ In recent years, hepatic arterial infusion pump (HAIP) chemotherapy has been further investigated.

The rationale for HAIP chemotherapy is primarily based on blood supply. Liver tumors, including iCCA and liver metastases, mainly derive their blood supply from the hepatic artery.^6,7^ During surgery, a catheter is placed in the gastroduodenal artery (GDA). The catheter is attached to a subcutaneous pump that infuses chemotherapy directly into the liver with a continuous flow of 1.5 cc per day. The agent used is floxuridine (FUDR), which is well-known for its high hepatic first-pass extraction. This allows for high tumor–drug concentrations and minimal systemic toxicity.^8,9^ Thus, the combination of these specific features of FUDR and an intra-arterial approach exposes iCCA to high-dose local chemotherapy while systemic exposure remains negligible.^10–12^

Various techniques for HAIP placement may be used—open, laparoscopic, and robotic. To date, only two retrospective single-center cohort studies compared perioperative outcomes of robotic and open HAIP placement for any treatment indication and showed that robotic placement is feasible.^13,14^ The limited evidence thus far favors robotic HAIP placement in experienced hands; however, no prospective or multicenter study has yet been performed. Additionally, other studies did not focus on patients with unresectable iCCA specifically, while surgery in these patients faces several technical obstacles, including substantial tumor size, involvement of the liver hilum or gallbladder, large positive hilar nodes, and inflammation because of biliary drainage.

Thus, this multicenter study aims to compare short-term outcomes of robotic HAIP placement with open HAIP placement in patients with unresectable iCCA in whom HAIP placement was performed after inclusion in the Dutch national PUMP-II trial.

## Methods

### Study Design and Patients

This multicenter cohort study compared robotic HAIP placement with open HAIP placement in patients with unresectable iCCA without extrahepatic disease. All patients were included from the PUMP-II trial (NL70452.078.19), a multicenter, phase II trial that assessed the effectiveness of HAIP chemotherapy in patients with unresectable iCCA. Surgical HAIP placements were performed according to the PUMP-II study protocol, from January 2020 to September 2022, in three academic hospitals: Amsterdam UMC, UMC Utrecht and Erasmus MC.^15^ Baseline patient and tumor characteristics, postoperative complications, re-admission, and mortality were prospectively obtained via the PUMP-II trial, while perioperative data, length of hospital stay (LOS), and time to functional recovery (TTFR) were retrospectively obtained from local electronic medical records by the local PUMP-II researchers (BTH, SF, RD, MR). A second assessment of all the data was performed by two of those researchers (BTH and SF), and in case of doubt, local electronic medical records were reassessed. Written informed consent was obtained from all participants for the PUMP-II trial.

### Primary Outcome

The primary outcome was TTFR reported in days. TTFR was reached as all of the following criteria were met: independently mobile at the preoperative level, adequate pain control with oral medication only, ability to maintain sufficient daily intake (at least 50% of required calories), absence of intravenous fluid administration, and no clinical signs of infection. In the case of suspected or known abdominal infection, this last item is met when the patient has no fever, and serum C-reactive protein concentration is decreasing and is below 150 mg/L.^16,17^

### Secondary Outcomes

Secondary outcomes were perioperative outcomes. The collection of these outcomes were divided into three main items. First, baseline patient and tumor characteristics were collected. This consisted of age, sex, body mass index (BMI), American Society of Anesthesiologists (ASA) score, prior chemotherapy treatment, prior abdominal surgery, aberrant arterial anatomy, placement of catheter tip, largest diameter of the primary tumor, and preoperative drainage. Second, perioperative outcomes were collected and these included estimated blood loss (EBL), duration of operation, and conversion rate. Lastly, postoperative data were collected, comprising LOS, time to start HAIP chemotherapy, readmission within 30 days, mortality within 90 days, postoperative complications within 90 days, and Clavien–Dindo classification grade III or higher (major complications). The postoperative complications were divided into catheter-related complications (hepatic artery dissection, hemorrhage at the site of catheter insertion, catheter dislocation) and other complications.

### General Perioperative Procedure

Prior to surgery, a multiphase contrast-enhanced computed tomography (CT) of the chest abdomen and pelvis was performed to evaluate disease extent and identify potential aberrant arterial anatomy to determine the position of the catheter. Patients were allocated to open or robotic HAIP placement primarily based on availability of the robot. All procedures were performed by experienced hepatobiliary surgeons (BGK, RW, JH, RJS). At all participating centers, both open and robotic HAIP placements were conducted. After surgery, a Technetium-99-labeled macro aggregated albumin nuclear medicine scan and perfusion CT scan was performed to rule out extrahepatic perfusion (EHP).

### Technique of Hepatic Arterial Infusion Pump (HAIP) Placement

HAIP placement was performed as an isolated procedure. Routinely, a concurrent cholecystectomy was performed during the pump placement to prevent the development of chemical cholecystitis caused by inadvertent intra-arterial chemotherapy of the gallbladder, but no additional liver resections were conducted in the same procedure.

### Standard Open Procedure of HAIP Placement

**Fig. 1:**
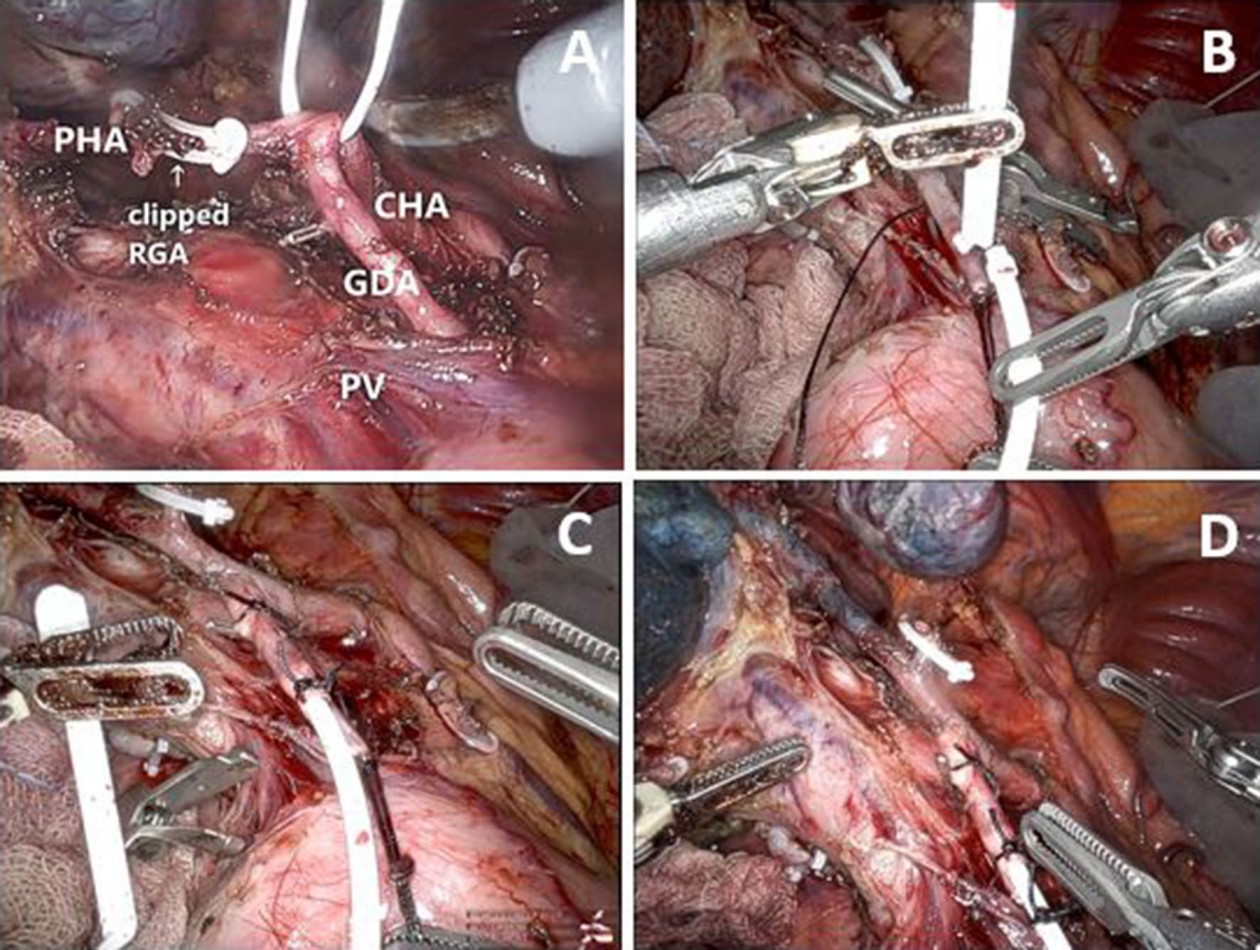
**A** Skeletonization of the common hepatic artery, proper hepatic artery and gastroduodenal artery. **B** Insertion of the catheter in the gastroduodenal artery. **C** Fixation catheter with three silk sutures. **D** Methylene blue dye test to test the presence of bilobar liver perfusion and absence of extrahepatic perfusion. *CHA* common hepatic artery, *PHA* proper hepatic artery, *GDA* gastroduodenal artery, *PV* portal vein, *RGA* right gastric artery

The following steps were conducted for open procedures. First, a laparoscopic abdominal exploration was conducted to rule out peritoneal metastases. Frozen sections were obtained if necessary. Abdominal access was then obtained using a subcostal incision. The hepatoduodenal ligament was dissected and a hilar lymphadenectomy was performed. A cholecystectomy followed. The ligament, and consequently the right gastric artery, were dissected to avoid perfusion of the stomach. The common hepatic artery (CHA), proper hepatic artery (PHA), and GDA were dissected to avoid EHP (Fig. 1a). Accessory and aberrant hepatic arteries were ligated to ensure the liver tumors receive only blood with FUDR. The distal GDA was ligated at 3–4 cm from the hepatic artery. A subcutaneous pump pocket was then created in the left abdomen and fixated with four silk ties. First, the catheter with beads was positioned intraperitoneally and then attached to the pump. Thereafter, vascular control was achieved by applying a bulldog clamp at the origin of the GDA or on the CHA and PHA. Two or three silk ties were positioned under the proximal GDA to later secure the catheter. A transverse arteriotomy of the anterior wall of the GDA, 2–3 cm from its origin, was performed. The catheter was then inserted into the GDA and positioned at its origin, where it meets the CHA, and tied with the two previously mentioned sutures (Fig. 1bc). Lastly, a methylene blue dye test was used to rule out EHP and confirm bilobar perfusion of the liver (Fig. 1d). In case of EHP, additional dissection in the ligament was performed. The catheter was flushed with heparinized saline and the wounds were closed.

### Robotic Procedure of HAIP Placement

The steps for open HAIP placement were similar for robotic placement with the exception of the method for intra-abdominal access. In robotic surgery, abdominal access was achieved using six ports: one 12 mm assistant port, four 8 mm ports for robotic arms and a camera, and one 5 mm port in the left upper quadrant for a liver retractor. Robotic HAIP placement was performed using the DaVinci Xi surgical system. A complete video demonstrating the robotic HAIP placement is provided as a supplement to this paper (Supplementary files 1 and 2).

### Statistical Analysis

Descriptive and comparative statistics were performed using SPSS for Windows version 24.0 (IBM SPSS Inc., Armonk, NY, USA). Continuous variables were expressed, dependent on distribution, as means and standard deviation (SD) or medians and interquartile ranges (IQRs) accordingly. A Student *t* test was performed for two-column parametric comparisons. A Mann–Whitney U test was performed for two-column, non-parametric comparisons. Categorical variables were expressed as frequencies and percentages using a Chi-square test or Fisher’s exact test. *P*-values were two-sided and a *p*-value <0.05 was considered statistically significant. A sensitivity analysis was conducted to assess the impact of the surgical site. All analyses were performed according to the intention-to-treat principle.

## Results

### Baseline Characteristics

Fifty patients were included, of whom 22 underwent robotic HAIP placement and 28 underwent open HAIP placement in three centers from January 2020 to September 2022. Each center performed six to eight robotic placements. The Erasmus MC performed 96% (27/28) of the open placements. Table 1 shows an overview of baseline characteristics of study participants. The median age was 63 years in the robotic group and 65 years in the open group. Aberrant arterial anatomy was seen in 50% (11/22) in the robotic group compared with 21% (6/28) in the open group (*p* = 0.042).

**Table 1:** Baseline characteristics of study participants

	Robotic	Open	*p*-Value
	[*n* = 22]	[*n* = 28]	
Age, years (median [IQR])	63 [55–71]	65 [57–72]	0.265
Female sex [*n* (%)]	15 (68)	20 (71)	1.000
Body mass index, kg/m^2^ (median [IQR])	25 [23–28]	25 [22–27]	0.584
ASA physical status [*n* (%)]			0.726
I	1 (5)	1 (4)	
II	14 (64)	15 (54)	
III	7 (32)	12 (43)	
Prior abdominal surgery [*n* (%)]	4 (18)	7 (25)	0.734
Additional systemic therapy [*n* (%)]	18 (82)	21 (75)	0.734
Tumor size on imaging, mm (median [IQR])	100 [72–116]	94 [73–124]	0.992
Aberrant arterial anatomy^a^ [*n* (%)]	11 (50)	6 (21)	0.042
Left hepatic artery	5 (23)	2 (7)	
Right hepatic artery	6 (27)	5 (18)	
GDA origin	2 (9)	0 (0)	
Catheter tip placement in GDA [*n* (%)]	22 (100)	27 (96)	1.000
Preoperative biliary drainage [*n* (%)]	2 (9)	5 (18)	0.444
University medical center			<0.001
Erasmus MC	8 (36)	27 (96)	
Amsterdam UMC	8 (36)	0 (0)	
UMC Utrecht	6 (27)	1 (4)	

^a^ Numbers may not add up due to multiple aberrant arteries

*ASA* American Society of Anesthesiologists, *GDA* gastroduodenal artery, *IQR* interquartile range

### Time to Functional Recovery (Primary Outcome)

The median TTFR was 2 [1–3] days after robotic HAIP placement and 5 [4–8] days after open HAIP placement (*p* < 0.001). Each sub-item of functional recovery was achieved earlier following robotic HAIP placement than following open HAIP placement (Table 2). All patients reached functional recovery within a maximum of 20 days. LOS was 3 [2–4] days in the robotic group versus 6 [4–9] days in the open group (*p* < 0.001). After conducting a sensitivity analysis for surgical site at Erasmus, given that 96% of the open placements were performed there, significant differences remained apparent: 2 days after robotic versus 5 days after open placement (*p* < 0.001) [Supplementary file 1].

**Table 2:** Time to functional recovery (primary outcome)

	Robotic	Open	*p*-Value
	[*n* = 22]	[*n* = 28]	
Time to functional recovery, days (median [IQR])	2 [1–3]	5 [4–8]	< 0.001
Restored mobility	2 [1–3]	4 [3–6]	< 0.001
Adequate pain control^a^	1 [1–2]	4 [3–6]	< 0.001
Adequate caloric intake^b^	1 [1–2]	4 [3–7]	< 0.001
No intravenous fluid administration	1 [1–2]	4 [3–5]	< 0.001
No signs of active infection	0 [0–1]	4 [3–5]	< 0.001
Length of hospital stay, days (median [IQR])	3 [2–4]	6 [4–9]	< 0.001
Readmission <30 days [*n* (%)]	3 (14)	5 (18)	1.000

^a^Oral analgesia only

^b^Minimum of 50% of required calories daily

*IQR* interquartile range

### Intraoperative Outcomes

Intraoperative outcomes are presented in Table 3. One patient (4.5%) in the robotic group underwent a conversion to open because of a large bulky tumor leaning on the hilum, immobilizing the liver. The median EBL was 100 [15–263] mL in the robotic group and 125 [100–375] mL in the open group (*p* = 0.110), and the median operative time was 233 [210–278] min and 220 [179–252] min, respectively.

**Table 3:** Intraoperative outcomes

	Robotic	Open	*p*-Value
	[*n* = 22]	[*n* = 28]	
Conversion to open [*n* (%)]	1 (5)	–	–
Estimated blood loss, mL (median [IQR])	100 [15–263]	125 [100–375]	0.110
Operative time, min (median [IQR])	233 [210–278]	220 [179–252]	0.125

*IQR* interquartile range

### Postoperative Outcomes

One of the patients in the open group died within 90 days after surgery due to a severe coronavirus disease 2019 (COVID-19) infection. The incidence of 90-day postoperative complications was comparable between both groups (Table 4). Specifically, 8/22 (36%) patients in the robotic group experienced a postoperative complication, versus 11/28 (39%) patients in the open group (*p* = 1.000). In both groups, one patient experienced a catheter-related complication, i.e. an hepatic artery thrombosis in the robotic group, and a combination of an hepatic artery dissection, EHP and hemorrhage at the catheter insertion site in the open group (*p* = 1.000).

**Table 4:** Postoperative complications within 90 days

	Robotic	Open	*p*-Value
	[*n* = 22]	[*n* = 28]	
Complications, all [*n *(%)]	8 (36)	11 (39)	1.000
Catheter-related complications [*n *(%)]	1 (5)	1 (4)	1.000
Hepatic artery thrombosis	1 (5)	0 (0)	
Extra hepatic perfusion^a^	0 (0)	1 (4)	
Hepatic artery dissection	0 (0)	1 (4)	
Hemorrhage (at catheter insertion site)	0 (0)	1 (4)	
Catheter dislocation	0 (0)	0 (0)	
Postoperative complications,^b^ other [*n *(%)]	8 (36)	11 (39)	1.000
Complications, Clavien–Dindo grade III or higher [*n* (%)]	4 (18)	6 (21)	1.000
IIIa	2 (9)	3 (11)	
IIIb	1 (5)	2 (7)	
IVa	1 (5)	0 (0)	
V	0 (0)	1 (4)	
HAIP chemotherapy after pump placement [*n* (%)]	22 (100)	26 (93)	0.497
Days to first HAIP chemotherapy (median [IQR])	14 [14–18]	18 [14–21]	0.153
90-day mortality [*n *(%)]	0 (0)	1 (4)	0.371

Percentages may not add up due to patients with multiple complications

^a^On postoperative nuclear scan

^b^Including pump-related complications and other complications

*HAIP* hepatic arterial infusion pump, *IQR* interquartile range

Major postoperative complications (Clavien–Dindo grade III or higher) occurred in 18% (4/22) of patients in the robotic group and 21% (6/28) of patients in the open group (*p* = 1.000). In the robotic group, these complications consisted of ascites, a wound infection, and a pulmonary embolism, and in the open group, these were pump-related complications, a bleeding of the GDA that required additional ligation, a small bowel perforation, an intra-abdominal collection. and postoperative cholangitis. Postoperative cholangitis occurred once in the robotic group and once in the open group. Both patients with cholangitis had undergone endoscopic retrograde cholangiopancreatography (ERCP) approximately 2 weeks prior to surgery.

All patients in the robotic group initiated HAIP chemotherapy following surgery, and this was accomplished in 93% (26/28) of patients in the open group (*p* = 0.497). The start of HAIP treatment in the open group was hindered twice. One patient passed away due to a severe COVID-19 infection, and the other patient experienced multiple catheter-related complications, including an hepatic artery dissection, making HAIP treatment impossible. The median time to start HAIP chemotherapy after pump placement was 14 [14–18] days in the robotic group and 18 [14–21] days in the open group (*p* = 0.153).

## Discussion

This study compared perioperative outcomes of robotic HAIP placement versus open HAIP placement in patients with unresectable iCCA. We found that TTFR was significantly shorter in patients who underwent robotic HAIP placement (2 vs. 5 days). Furthermore, all patients in the robotic group and 93% (26/28) of patients in the open group were able to receive HAIP chemotherapy following the surgical procedure. One patient was unable to receive HAIP treatment because of catheter-related complications. Intra- and postoperative complications were similar in both groups.

As this study represents the first study comparing robotic and open HAIP placements in patients with iCCA, our results can only be compared with studies evaluating HAIP placements in patients with colorectal liver metastases (CRLM). Our primary outcome was TTFR since it is considered a more accurate reflection of physical recovery than LOS. Unfortunately, since no prior studies investigating robotic HAIP placements have employed TTFR as an outcome measure, direct comparisons cannot be made. LOS has frequently been used as an outcome measure.

In our cohort, a significant difference in LOS was observed in favor of robotic surgery (3 days vs. 6 days; *p* < 0.001). This finding contrasts with that of Qadan et al., who reported no difference in LOS (5 days vs. 4 days).^13^ Dhir et al. reported that LOS was 4.5 days after robotic placement in patients with CRLM (17 patients) and iCCA (4 patients), while Allen et al. observed an LOS of 11 days after open placement in patients with CRLM.^14,18^ This difference in LOS, compared with our cohort, might be due to concomitant liver or colon resections in the published cohorts. It is important to mention that LOS can be influenced by various external factors. In this study, certain external factors, such as standard clinically planned postoperative perfusion scintigraphy scans on postoperative day 3 or 4, might have contributed to slightly prolonged hospital stays.

Catheter-related complications were comparable, occurring in one patient in each group (5% vs. 4%). Allen et al. conducted a study evaluating 544 open HAIP placements and reported a similar catheter-related complication rate of 6%.^18^ Due to the catheter-related complications, one patient in the open group was unable to start HAIP chemotherapy. In total 93% (26/28) of patients in the open group were able to initiate HAIP chemotherapy following the surgical procedure. The inability of the other patient in the open group to initiate HAIP chemotherapy was because of a severe COVID-19 infection. All patients in the robotic group were able to start HAIP chemotherapy.

Other postoperative complications were similar in both groups. All patients were primarily eligible for a robotic approach, with an open approach employed only when the robot was unavailable. Conversion to an open procedure was performed once (5%) because of a large bulky tumor leaning on the hilum, immobilizing the liver. The conversion rates in other cohorts ranged from 4% to 13%, most likely impacted by the presence of concomitant resections.^13,14^ When evaluating major complications using Clavien–Dindo grade 3 or higher, Qadan et al. demonstrated complication rates of 23% in the open group and 18% in the robotic group among patients with CRLM, which aligns with our findings of 21% and 18%, respectively.^13^ One patient in each group experienced cholangitis. This was associated with preoperative endoscopic drainage rather than a postoperative complication since both patients had undergone ERCP approximately 2 weeks prior to surgery.

Regarding intraoperative outcomes, no significant difference was observed in EBL between the two groups in our cohort (100 mL vs. 125 mL). Thus far, no other studies have reported significant differences in EBL. Since the HAIP placement is a relatively simple procedure, no excessive peroperative blood loss was expected in any of the two groups. The mean operation time in our cohort was similar in both groups (233 min vs. 220 min; *p* = 0.125). In contrast, Qadan et al. demonstrated a significant difference in operation time for HAIP placement without concomitant resection, with a mean operation time of 272 min in the robotic group and 154 min in the open group (*p* < 0.01). Operation time in the present study was probably longer in both groups because HAIP chemotherapy was still being introduced in The Netherlands.

Utilization of the robotic platform might also result in increased operational costs due to extended procedure durations and the use of more expensive instrumentation.^19^ However, since in this study no differences in operation duration were observed, robotic placement may not only contribute to a clinically significant acceleration of recovery by 60% (2 vs. 5 days) but may also potentially lead to cost reductions resulting from shorter hospital stays and comparable operative time.

This study possesses numerous strengths. First, it represents the first study comparing open and robotic HAIP placements using data from a prospective study. Second, the choice of TTRF as the primary outcome ensures a valid and clinically relevant measurement. Limitations include a small sample size, which may restrict statistical power to determine small differences between the groups. Additionally, the allocation of patients to either robot or open placement was not randomized; instead, the surgical approach was determined based on availability of the robot.

## Conclusion

Robotic placement of HAIP in patients with unresectable iCCA is safe and results in a significant reduction in the TTFR. These findings may lower the barrier to HAIP chemotherapy since a laparotomy is not required with robotic placement.

### Supplementary Information

Below is the link to the electronic supplementary material.

Supplementary file1 (DOCX 26 KB)

Supplementary file2 (MP4 345986 KB)
