# Supplementary material for: Robotic Versus Open Hepatic Arterial Infusion Pump Placement for Unresectable Intrahepatic Cholangiocarcinoma
Source: Ann Surg Oncol. 2024 Mar 18;31(6):4022–9. doi: 10.1245/s10434-024-15127-w (PMC11076355; doi:10.1245/s10434-024-15127-w)
Supplement: Supplementary file 1 — Supplementary file1 (DOCX 26 KB) [file 10434_2024_15127_MOESM1_ESM.docx]

**Supplementary Material**

**Appendix 1. Video_1.** Robotic HAIP pump placement. **Hyperlink:** [Video Robotic HAIP placement](https://eur04.safelinks.protection.outlook.com/?url=https%3A%2F%2Fvimeo.com%2F844976067%3Fshare%3Dcopy&data=05%7C01%7Cb.tenhaaft%40amsterdamumc.nl%7C4cb6505a7350454d6cf408db83b0a142%7C68dfab1a11bb4cc6beb528d756984fb6%7C0%7C0%7C638248566854531689%7CUnknown%7CTWFpbGZsb3d8eyJWIjoiMC4wLjAwMDAiLCJQIjoiV2luMzIiLCJBTiI6Ik1haWwiLCJXVCI6Mn0%3D%7C3000%7C%7C%7C&sdata=jmvT9WjNKZwE%2FrhvQwR3Pv6eY1QAa0JO9ZhLQ1OASDI%3D&reserved=0) 

Password: haipump

**Appendix 2. Sensitivity analysis of Time to Functional Recovery for surgical site at Erasmus MC**

|  |  |  |  |
| --- | --- | --- | --- |
| **Table 5. Time to Functional Recovery (Erasmus MC)** | | |  |
|  | **Robotic** | **Open** | ***P*** |
|  | **n=8** | **n=27** |  |
| Time to functional recovery, median [IQR], days | 2 [1-3] | 5 [4-8] | <0.001 |
| Restored mobility | 2 [1-2] | 4 [3-6] | <0.001 |
| Adequate pain control* | 2 [1-2] | 4 [3-6] | <0.001 |
| Adequate caloric intake^+^ | 1 [1-2] | 4 [3-7] | <0.001 |
| No intravenous fluid administration | 1 [1-3] | 4 [3-5] | <0.001 |
| No signs of active infection | 1 [1-1] | 4 [3-5] | <0.001 |
| Length of hospital stay, median [IQR], days | 2 [1-3] | 6 [4-9] | <0.001 |
| *Oral analgesia only. ^+^Minimum of 50% of required calories daily. | | | |
|  |  |  |  |
